# Efficacy and Safety of Boldine Combined with *Phyllanthus niruri* and *Ononis spinosa* in Medical Expulsive Therapy for Distal Ureteral Stones with Renal Colic: A Single-Center, Retrospective Cohort Study

**DOI:** 10.3390/medicina60091455

**Published:** 2024-09-05

**Authors:** Ernesto Di Mauro, Pietro Saldutto, Roberto La Rocca, Giuseppe Sangiorgi, Gianluigi Patelli, Biagio Barone, Vittore Verratti, Roberto Castellucci, Luigi Napolitano, Fabrizio Iacono, Vincenzo Maria Altieri

**Affiliations:** 1 Urology Unit, Department of Neurosciences, Reproductive Sciences and Odontostomatology, University of Naples “Federico II”, 80138 Naples, Italy; ernestodm9@gmail.com (E.D.M.); robertolarocca87@gmail.com (R.L.R.); 2 Department of Urology, Humanitas Gavazzeni, 24125 Bergamo, Italy; pietrosaldutto@gmail.com; 3 Department of Biomedicine and Prevention, University of Rome Tor Vergata, 00133 Rome, Italy; gsangiorgi@gmail.com; 4 Radiology Department, ASST Bergamo Est, 24068 Seriate, Italy; gianluigi.patelli@gmail.com; 5 Department of Urology, Ospedale del Mare, ASL NA1 Centro, 80147 Naples, Italy; biagio.barone@unina.it; 6 Department of Psychological, Health and Territorial Sciences, University “G. d’Annunzio” Chieti-Pescara, 66100 Chieti, Italy; vittore.verratti@unich.it; 7 Department of Urology, SS. Annunziata Hospital, ASL 2 Abruzzo, 66100 Chieti, Italy; roberto.castellucci@gmail.com; 8 Department of Medicine and Health Sciences “V. Tiberio”, University of Molise, 86100 Campobasso, Italy; info@fabrizioiacono.it (F.I.); vincenzomaria.altieri@gmail.com (V.M.A.)

**Keywords:** kidney stone, boldine, MET, urolithiasis

## Abstract

*Background and Objectives*: This study aimed to compare the effects and safety of boldine combined with *Phyllanthus niruri* and *Ononis spinosa* plus tamsulosin vs. tamsulosin alone in medical expulsive therapy (MET) for distal ureteral calculi. *Materials and Methods*: This retrospective cohort study was conducted on 159 renal colic patients with distal ureteric stones (≤10 mm). Patients aged between 18 and 70 years or older with distal ureteral (below the sacroiliac joint) stones ≤10 mm (defined by the largest diameter in three planes) confirmed by urinary ultrasonography and/or native computed tomography (CT). Patients were divided into two groups: A and B. Patients in Group A received tamsulosin 0.4 mg plus boldine combined with *Phyllanthus niruri* and *Ononis spinosa*, while those in Group B received tamsulosin 0.4 mg. The rate of stone expulsion, duration of stone expulsion, the dose and the duration of nonsteroidal anti-inflammatory drugs (NSAIDs), analgesic use, and adverse effects of drugs were recorded. *Results*: No differences were reported in demographic profiles between the two groups. The stone expulsion rate in Group A (84.8%) was higher in comparison to Group B (52.5%); the mean time of stone expulsion was 16.33 ± 4.75 days in Group A and 19.33 ± 6.42 days in Group B. The mean requirement time of analgesia was significantly less in Group A, 2.42 ± 2.56, than in Group B, 6.25 ± 3.05. Drug-related adverse effects (headache, dizziness, nausea, vomiting, postural hypotension, backache, and running nose) were comparable between the two groups. *Conclusions*: Tamsulosin plus boldine combined with *Phyllanthus niruri* and *Ononis spinosa* as medical expulsion therapy is more effective for distal ureteric stones with less need for analgesics and a shorter stone expulsion time than tamsulosin alone.

## 1. Introduction

Urolithiasis is one of the most prevalent medical conditions worldwide, with a lifetime prevalence ranging from 1% to 15%. This condition affects millions of people across the world and presents a peak age of incidence typically occurring around 30 years [1]. Urolithiasis is not only widespread but also exhibits a significant gender disparity, affecting men two to three times more frequently than women. The formation of stones in the urinary tract is a complex process influenced by various factors, including diet, metabolism, infections, hereditary predispositions, congenital anatomical defects, environmental influences, and systemic disorders [2]. Among the different types of stones, ureteral stones are particularly common, accounting for approximately 20% of all cases of urolithiasis. Interestingly, about 70% of these ureteral stones are located in the distal third of the ureter, which is the portion closest to the bladder [3]. This specific location of the stones has significant implications for their treatment and potential for spontaneous passage. It is noteworthy that approximately 50% of ureteral stones eventually pass spontaneously without the need for surgical intervention. The likelihood of spontaneous passage is primarily determined by the size of the stone, with smaller stones being more likely to pass on their own [4]. The prevalence of urinary tract stones is higher in industrialized nations, which may be attributed to differences in diet, lifestyle, and environmental factors [5]. The composition of urine plays a crucial role in the formation of stones, with certain dietary habits and metabolic conditions increasing the risk of stone formation. The most common types of renal stones are composed of calcium oxalate, followed by calcium phosphate, uric acid, and struvite. Each type of stone has its unique characteristics and implications for treatment, making accurate identification essential for selecting the most appropriate therapeutic approach [6,7,8]. In recent years, significant advancements have been made in the treatment of ureteral stones, with both surgical and medical options available to patients [9]. One of the most established medical treatments for distal ureteral stones, particularly those less than 10 mm in size, is medical expulsive therapy (MET) [10]. The primary goal of MET is to facilitate the passage of stones, thereby avoiding the need for more invasive surgical procedures. This is achieved by using medications that relax the smooth muscles of the ureter, reduce peristaltic activity, and ultimately increase the rate of stone expulsion [11]. Among the various agents used in MET, alpha-1 adrenergic blockers, such as tamsulosin, are the most widely used. Tamsulosin works by relaxing the smooth muscles of the ureter, which helps to reduce the discomfort associated with stone passage and increases the likelihood of successful stone expulsion [12]. In addition to alpha-1 adrenergic blockers, newer classes of medications have also been introduced in the management of urolithiasis. Phosphodiesterase-5 (PDE5) inhibitors have been explored for their potential benefits in treating ureteral stones. These inhibitors regulate muscle contraction and relaxation through the metabolism of cyclic nucleotides, such as cyclic guanosine monophosphate (cGMP) and cyclic adenosine monophosphate (cAMP). By influencing these pathways, PDE5 inhibitors may offer an additional mechanism for promoting stone passage [13]. In the last few decades, there has been a growing interest in the use of plant-derived bioactive compounds in the treatment of various diseases, including urolithiasis [14]. One of the most well-known plants in this regard is *Phyllanthus niruri* (PN), commonly referred to as the “stonebreaker”. PN has been used traditionally for its potential benefits in managing kidney stones, and recent studies have supported its role in interfering with different stages of stone formation [15]. PN appears to inhibit crystal aggregation, alter stone structure and composition, and promote ureteral relaxation. Additionally, it has been shown to reduce calcium excretion, which is a key factor in the formation of calcium-based stones [16]. Another plant-derived compound that has gained attention for its potential in treating urolithiasis is boldine. Boldine is known for its diverse biological effects, including antioxidant, anti-inflammatory, anti-epileptic, and neuroprotective properties. Recent research has also suggested that boldine may be effective in the treatment of urinary stones, particularly through its role in lithotripsy [17]. Furthermore, boldine has been reported to act as an alpha-1 adrenoceptor blocker, which may contribute to its effectiveness in promoting stone passage [18]. In light of these developments, a new nutraceutical formulation has been developed, combining *Phyllanthus niruri*, *Ononis spinosa*, and boldine. This formulation aims to harness the synergistic effects of these plant-derived compounds in the management of distal ureteral stones. The present study was designed to evaluate the safety and efficacy of this novel nutraceutical combination, in conjunction with tamsulosin, as a medical expulsive therapy for distal ureteral stones. The study’s findings have the potential to offer a new, effective treatment option for patients suffering from urolithiasis, particularly those with distal ureteral stones who wish to avoid more invasive interventions.

## 2. Materials and Methods

**Figure 1:**
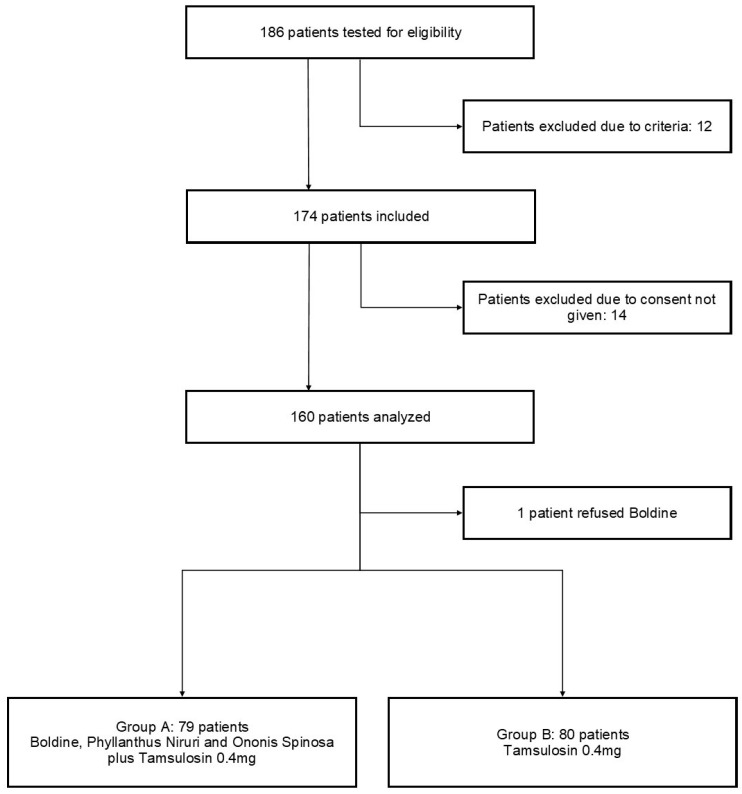
Flow chart of patients included in the study.

We conducted a retrospective cohort study at the tertiary care hospital, Humanitas Gavazzeni, in Bergamo, Italy, between March 2023 and December 2023. The study received approval from the Institutional Review Board of Humanitas Gavazzeni, Bergamo, Italy (protocol number 02/24 GAV). All procedures adhered to the ethical guidelines of the Helsinki Declaration, and all participants provided written informed consent prior to enrollment in the study. The inclusion criteria for the study were carefully defined to ensure the selection of appropriate participants. Eligible patients were adults over the age of 18 years who presented with distal ureteral stones, specifically located below the sacroiliac joint. The stones had to measure less than 10 mm in their largest diameter as confirmed by either urinary ultrasonography or non-contrast computed tomography (CT). Additional criteria included the submission to extracorporeal shock wave lithotripsy with a single residual ureteral stone fragment. Patients were excluded if they were currently using alpha-adrenoceptor antagonists, as these medications could interfere with the study’s outcomes. Other exclusion criteria included the presence of a urinary tract infection, severe refractory pain, hydronephrosis, acute or chronic renal failure, multiple ureteral stones, bilateral ureteral calculi, or the presence of a single functioning kidney. Patients with any history of ureteral surgery or procedures, or urinary tract anomalies such as a horseshoe kidney or duplex urinary system, were also excluded. Furthermore, pregnant or lactating women, individuals with urethral stricture or a history of ureteral strictures, patients with diabetes mellitus, those with hypotension (defined as a systolic blood pressure below 100 mmHg), current users of corticosteroids, and anyone with known or suspected allergies to the study medications were not eligible to participate. A total of 186 patients who met the criteria were enrolled in the study. Patients were assigned to groups based on chronological criteria: those treated earlier in the study were assigned to Group A and received boldine combined with *Phyllanthus niruri* and *Ononis spinosa* plus one capsule of tamsulosin 0.4 mg daily; those treated later were assigned to Group B and received tamsulosin 0.4 mg alone daily, until spontaneous stone passage, up to a maximum of 28 days or the need for intervention (Figure 1). The patients were followed up for 28 days and then for a total follow-up of 3 months. At the screening visit, patients underwent clinical examination and the following data were collected: age, sex, stone side, and stone size. The stone expulsion time, analgesic use, number of hospital visits for pain, and adverse effects of drugs were noted. Patients were instructed to drink up to 2 L of water daily and to filter their urine with a thin cloth or net to detect stone expulsion. For pain control during colicky episodes, 50 mg sodium diclofenac suppositories were used on an as-needed basis.

### 2.1. Outcomes

The primary endpoint was the stone expulsion rate, defined as stone expulsion, confirmed by negative findings on an ultrasound scan or CT over the 28 d surveillance period. The secondary endpoints were the stone expulsion time, number of colicky attacks, analgesics required, and drug side effects. Patients who failed to pass the stone after 28 days were subjected to active treatment.

### 2.2. Statistical Analysis

Data were collected by filling in pro forma data sheets, which included the patients’ demographic profiles, investigation reports, and the results of primary and secondary outcomes. Continuous data were expressed as means and standard deviations, while categorical data were expressed as frequencies and percentages. The normal distribution of data was assessed via the Kolmogorov–Smirnov test. Data were analyzed by using SPSS, ver. 17.0 (SPSS Inc., Chicago, IL, USA). Pearson’s chi-squared test was utilized for categorical data, while an independent-samples Mann–Whitney U test was utilized for continuous variables. All statistical tests were based on two-tailed probability, and a *p*-value < 0.05 was considered statistically significant.

## 3. Results

Out of the 186 patients initially assessed for eligibility, 174 were deemed suitable for inclusion in the study. Fourteen patients were excluded due to not providing consent, and one patient refused to participate in the medical expulsive therapy (MET) involving boldine. Consequently, the final analysis included data from 159 patients, with 79 in the treatment group (Group A) and 80 in the control group (Group B).

The demographic characteristics of the study population were similar between the two groups. In Group A, the mean age of patients was 59.95 ± 17.16 years, while in Group B, the mean age was 56.03 ± 12.28 years. The overall gender distribution across both groups was nearly equal, with 49.7% of the patients being male and 50.3% female. Both groups were also comparable in terms of other baseline characteristics, including body mass index (BMI), duration of symptoms, and stone parameters such as size and laterality (Table 1). The primary outcome of the study, the stone expulsion rate, showed a significant difference between the two groups. In Group A, which received the combination therapy of boldine, *Phyllanthus niruri*, *Ononis spinosa*, and tamsulosin, the stone expulsion rate was 84.8%. In contrast, Group B, which received tamsulosin alone, had a significantly lower expulsion rate of 52.5% (*p* < 0.0001). Additionally, the average time to stone expulsion was shorter in Group A, with a mean duration of 16.33 ± 4.75 days compared to 19.33 ± 6.42 days in Group B. Despite the overall success in stone expulsion, there were instances where the stones were not expelled within the initial treatment period. In Group B, 38 patients (47.5%) failed to expel the stones, compared to 12 patients (15.2%) in Group A. For these patients, the medical treatment was extended for an additional two weeks. Even with the extended treatment, a subset of patients still required surgical intervention due to the failure of medical management. Specifically, 10 patients in Group B (12.6%) and 4 patients in Group A (5%) ultimately underwent surgery. Another finding of the study was the requirement for analgesic use during the treatment period. Patients in Group A required significantly less analgesia, with a mean usage time of 2.42 ± 2.56 days, compared to 6.25 ± 3.05 days in Group B (Table 2). Regarding drug-related adverse effects, the incidence was relatively low and comparable between the two groups. Reported side effects included headache, dizziness, nausea, vomiting, postural hypotension, backache, and runny nose. Nausea was observed in two patients in Group A, while postural hypotension was reported in three patients in Group A and two in Group B. Overall, the side effects were manageable and did not significantly impact the patients’ ability to continue the treatment.

## 4. Discussion

To the best of our knowledge, this study is the first to assess the efficacy and safety of tamsulosin 0.4 mg combined with boldine, *Phyllanthus niruri*, and *Ononis spinosa* in the context of medical expulsive therapy (MET). In recent decades, various studies have investigated the efficacy of different drugs and natural herbal medicines in managing urolithiasis, reflecting an ongoing interest in optimizing treatment strategies for stone expulsion. The European Association of Urology (EAU) guidelines highlight the benefits of MET in reducing the risk of intraoperative ureteral injury, accelerating the passage of stone fragments, and decreasing the frequency of colic episodes compared to surgical intervention guidelines reinforce the importance of exploring and validating non-invasive methods for managing ureteral stones [19]. Tamsulosin, an alpha-1 adrenergic receptor blocker, has been widely studied and is known to be effective in facilitating the passage of ureteral stones. Ibrahim et al. reported that tamsulosin was associated with a higher stone expulsion rate compared to alfuzosin, with rates of 85% and 75%, respectively. This underscores tamsulosin’s role as a cornerstone in MET [20]. A meta-analysis by Cui et al., which included 56 randomized controlled trials (RCTs) and 9395 patients, further supported the efficacy of tamsulosin 0.4 mg. The analysis revealed that tamsulosin was associated with a higher stone expulsion rate (RR 1.44, 95% CI 1.35–1.55, *p* < 0.01), a shorter stone expulsion time (weighted mean difference −0.73, 95% CI −1.00 to −0.45, *p* < 0.01), and fewer instances of surgical intervention (RR 0.68, 95% CI 0.50–0.93, *p* = 0.017), particularly in stones larger than 5 mm [21]. More recently, PDE5i has been also explored in MET. PDE5i has shown direct effects on the relaxation of ureteral smooth muscle, influencing the frequency of peristaltic waves and overall muscle tone. A systematic review by Cardona et al., which included four studies and 580 patients, suggested that PDE5i could be an effective treatment in MET. However, the review emphasized the need for more high-quality trials to validate these findings [22]. Natural herbal medicines, known for their multi-component, multitarget, and multi-pathway effects, have also been studied for their potential in stone management. *Phyllanthus niruri* is one such herb that has shown promise in interfering with crucial stages of calculi formation, including crystal structure and composition [15]. *Phyllanthus niruri* contains triterpenes, which are considered an important anti-lithogenic factor [23]. These compounds reduce the urinary excretion of oxalate and calcium, while also interfering with glycosaminoglycans in the matrix of precipitating crystals, making the crystals smoother and more fragile. This property of *Phyllanthus niruri* suggests that it could play a significant role in the prevention of lithiasis, inhibiting calculus growth and facilitating the dispersion and easier elimination of crystals in urine [24]. In addition to its anti-lithogenic properties, *Phyllanthus niruri* has been reported to promote ureteral relaxation and reduce the excretion of promoters of urinary crystallization such as calcium. This is particularly beneficial following lithotripsy, where it can aid in clearing residual fragments. Micali et al. reported that self-administration of *Phyllanthus niruri* after extracorporeal shock wave lithotripsy significantly increased the stone-free rate, particularly for stones located in the lower calyces [25]. Furthermore, Pucci et al. demonstrated that supplementation with *Phyllanthus niruri* increased the magnesium/creatinine and potassium/creatinine ratios, both of which are protective against stone formation. Notably, *Phyllanthus niruri* is effective and well-tolerated, with few side effects reported [26]. Boldine, another plant-derived compound, has selective activity on alpha-1 adrenergic receptor subtypes and possesses renal vasodilatory properties. Boldine, an aporphine alkaloid derived from the leaves of *Peumus boldus*, offers a range of beneficial effects, including improved endothelial function, blood pressure regulation, antioxidant properties, cytoprotective abilities, and anti-inflammatory and antiproliferative characteristics. These properties suggest that boldine could be a valuable component of MET, particularly in its ability to reduce damage in kidney diseases and facilitate the passage of ureteral stones [27]. Ureteral peristalsis and ureteral smooth muscle relaxation represent an important issue in MET. Ureteral peristalsis is regulated by interstitial cells of Cajal (ICC)-like cells, which represent the renal pacemaker cells [28]. Recently, the literature has shown the expression of different receptors along the ureter, particularly in the distal one-third of ureter, with different pharmacology effects [29]. In particularly α_1A_-adrenoceptor subtypes seem to be involved in contractile responses [30]. In fact, the inhibition of these receptors reduces the ureteral basal tone and increases the intra-luminal ureteral pressure. *P. niruri* showed an important activity in ureteral motility: Calixto et al. reported that alkaloids extracted from *P. niruri* present an antispasmodic activity, due to smooth muscle relaxation [31]. In fact, they reported that an alkaloid called ALK-1 was able to induce smooth muscle relaxation. Recently, Maisto et al. reported the myorelaxant effects of a nutraceutical formulation containing PN on human pulmonary artery smooth muscle cells (HPASMCs). The NF induced a decreased muscle contractility of −49.4% (*p* < 0.01) compared to the control [14]. According to Ivorra et al., boldine has procynetic and diuretic effects on kidneys and ureters and stimulates peristalsis of the urinary musculature [32]. The muscle relaxation increases the chance of stone passage and reduces the time to expulsion.

*Ononis spinosa* is a flowering shrub native to the Mediterranean, Asia, and Africa, traditionally used for various health problems, including kidney and bladder diseases. It has demonstrated antibiotic, antifungal, antipyretic, anti-inflammatory, antiseptic, and diuretic effects. Historically, it has been widely used in the treatment of rheumatism, urinary tract infections, and skin diseases [33]. Bashan et al. reported that *Ononis spinosa* exhibits a direct litholytic effect on kidney stones, particularly those composed of uric acid. Additionally, Addotey et al. found that a dichloromethane extract from *Ononis spinosa* has anti-hyaluronidase activity, which increases diuresis and could contribute to stone expulsion [34,35]. In the present study, the stone expulsion rate was significantly higher in the group receiving the combination therapy (84.8% vs. 52.5%) compared to the group receiving tamsulosin alone. The three herbal extracts—boldine, *Phyllanthus niruri*, and *Ononis spinosa*—appear to work synergistically to stimulate ureteral contractions and peristalsis, reduce the adhesivity of crystalloids, and promote stone expulsion. These multifactorial activities suggest that such nutraceutical products can interact with different stages of stone formation and expulsion, offering an alternative or complementary treatment to traditional pharmacotherapies for urolithiasis. This approach could potentially reduce treatment costs and minimize side effects associated with conventional therapies. While this study is pioneering in assessing the efficacy and safety of combining boldine, *Phyllanthus niruri*, and *Ononis spinosa* with tamsulosin in MET for distal ureteral calculi, it is important to acknowledge the study’s limitations. First, the retrospective and single-center nature of the study may introduce biases related to patient selection and treatment outcomes. Second, the relatively small sample size limits the statistical power of the findings and may affect the generalizability of the results. Third, the extensive exclusion criteria, although necessary for ensuring a homogeneous study population, may limit the applicability of the findings to the broader population of patients with ureteral stones. To address these limitations and build on the promising results of this study, further research is needed. Future studies should aim to conduct randomized, prospective, double-blind, placebo-controlled trials with larger sample sizes. Such trials would provide stronger evidence to support the use of this combination therapy in clinical practice. Additionally, exploring the mechanisms of action of these herbal extracts in greater detail could offer insights into how they can be optimized for use in MET and other therapeutic applications.

## 5. Conclusions

This study provides valuable preliminary evidence that combining boldine, *Phyllanthus niruri*, and *Ononis spinosa* with tamsulosin may serve as an effective and safe alternative to conventional MET for treating distal ureteral calculi. The multifaceted effects of these herbal compounds, particularly in promoting ureteral relaxation and facilitating stone expulsion, highlight their potential significance in the future management of urolithiasis. However, confirming these findings through further research is essential.

## Figures and Tables

**Table 1:** Demographic characteristics.

Group	Group A	Group B	*p*-Value
Age (Mean ± SD)	59.95 ± 17.169	56.03 ± 12,286	0.069
BMI (Mean ± SD)	26.81 ± 3.71	26.18 ± 3.67	0.217
Male/Female	47/32	32/48	0.071
Stone size, mm (Mean ± SD)	7.49 ± 2.46	6.79 ± 2.49	0.109
Stone side, *n*% Right/Left	53%/47%	45%/55%	0.087
Serum creatinine, mg/dL	1.2 ± 0.3	1.3 ± 0.35	0.124

**Table 2:** Clinical outcomes of patients.

Group	Group A	Group B	*p*-Value
Number of colicky episode (Mean ± SD)	1.01 ± 0.67	2.40 ± 1.22	<0.001
Pain score (1–10) (Mean ± SD)	4.15 ± 1.27	6.91 ± 1.22	<0.001
Expulsion of stone time, days (Mean ± SD)	16.33 ± 4.75	19.33 ± 6.42	<0.001
Doses of used NSAID mg (Mean ± SD)	1242.11 ± 938.45	2363.75 ± 953.77	<0.001
Analgesic requirement time, days (Mean ± SD)	2.42 ± 2.56	6.25 ± 3.05	<0.001

## Data Availability

The data presented in this study are available upon request from the corresponding author. The data are not publicly available due to privacy restrictions.
